# Structural Mutations Set an Equilibrium Noncoding Genome Fraction

**DOI:** 10.1093/molbev/msaf315

**Published:** 2025-11-28

**Authors:** Juliette Luiselli, Paul Banse, Olivier Mazet, Nicolas Lartillot, Guillaume Beslon

**Affiliations:** INSA-Lyon, CNRS, Université Claude Bernard Lyon 1, ECL, Université Lumière Lyon 2, LIRIS UMR5205, Lyon F-69621, France; BioTiC Team, Inria Lyon La Doua, Villeurbanne, France; Institute for Socio-Economic, University of Duisburg-Essen, Duisburg, Germany; Center for Economics and Neuroscience, University of Bonn, Bonn, Germany; Institut National des Sciences Appliquées, Institut de Mathématiques de Toulouse, Université de Toulouse, Toulouse 31062, France; Université Claude Bernard Lyon 1, Laboratoire de Biométrie et de Biologie Évolutive UMR CNRS 5558, Université Lyon 1, Villeurbanne, France; BioTiC Team, Inria Lyon La Doua, Villeurbanne, France; INSA-Lyon, INRIA, CITI EA3720, Lyon F-69621, France

**Keywords:** evolution, noncoding genome, genome size, mathematical modeling, structural mutations

## Abstract

The evolution of noncoding genome size remains poorly understood. While part of noncoding DNA arguably plays a regulatory role, a significant portion does not appear to have a detectable phenotypic effect. The abundance of nonfunctional DNA in genomes, observed across the Tree of Life, challenges purely adaptationist explanations. Several nonadaptive theories have been proposed to explain its presence and identify its determinants, emphasizing either the mutational processes or the mutational hazard entailed by noncoding and nonfunctional DNA. However, those theories have not yet been integrated into a common framework, and the exact nature of the mutational hazard is not yet fully understood. In this work, we introduce a simple mathematical model of genome size evolution. Our model shows that the noncoding fraction of the genome is shaped by two fundamental forces: (i) inherent biases in mutational neutrality—adding base pairs being more likely to be neutral than removing some and (ii) robustness selection arising from the mere existence of structural mutations—larger genomes being more prone to double-strand breaks that generate such mutations, thereby imposing a second-order selection on robustness. Together, these forces establish an equilibrium noncoding fraction that depends solely on mutation biases and the product of population size and mutation rate.

## Introduction

Genome size varies greatly throughout the Tree of Life: from $10^{5}$ base pairs (bp) for some bacteria (Riley et al. 2017), to more than $10^{11}$ bp for some plants (Pellicier et al. 2010). Coding sequences contribute to this variation through adaptive changes, but some parts of the genome seem devoid of phenotypic function and yet are highly variable in size (Liu et al. 2013). While noncoding DNA contains functional sequences, including regulatory regions (Rinn and Chang 2012), large stretches seem to bear no function whatsoever. This “junk” DNA (Ohno 1972; Doolittle 2013; Palazzo and Gregory 2014; Fagundes et al. 2022) is ubiquitous in all domains of life, regardless of genome sizes (Ahnert et al. 2008) and Gil and Latorre (2012). However, there is currently no consensus on the reasons behind the existence and maintenance of junk DNA (Fagundes et al. 2022).

In this work, we address the determinants of the amount of noncoding nonfunctional DNA. Several hypotheses have been proposed to address these issues, notably reviewed in Blommaert (2020).

In adaptive hypotheses, genome size itself is under selection due to its phenotypic impact on *e.g.* nucleus size or replication time (Malerba et al. 2020). In this view, genome size would be selectively limited (Kang et al. 2015; Bales and Hersch-Green 2019). Furthermore, the position of genes relative to each other or the centromere influences their expression (El Houdaigui et al. 2019). As such, it represents a potential selective pressure on the amount of intergenic DNA (Freeling et al. 2015). However, it can be argued that the variation in the proportion of noncoding DNA between species might be too high to be explained by these mechanisms (Petrov 2002; Blommaert 2020). More fundamentally, there is little direct evidence that selection induced by these phenotypes is strong enough to modulate the fate of mutations changing genome size.

Nonadaptive hypotheses have also been developed to decipher the mechanisms by which noncoding DNA could vary and stabilize. First, mutational explanations emphasize the impact of mutational patterns on the long-term evolution of genome size. In particular, the mutational equilibrium hypothesis (MEH) (Petrov 2002) suggests that two different mutational biases of opposite directions—a negative bias on short indels and a positive long insertion/deletion bias that decreases with genome size—could mechanistically explain the existence of an equilibrium genome size. The equilibrium itself would be modulated between species by the variation in the strength of those biases.

The mutational hazard hypothesis (MHH) (Lynch and Conery 2003), on the other hand, proposes an explanation in terms of *fixation* biases acting on mutations, related to second-order selective effects. According to the MHH, the noncoding genome expands by mutation, drift, and the insertion of selfish elements. However, this expansion increases the number of targets for deleterious mutations—*e.g.* such as gain-of-function mutations or loss of accurate splicing (Lynch 2007). In other words, noncoding DNA presents a mutational liability. As a result, genome expansion entails a slight selective cost, which could provide a sufficient force counteracting the growth of genome size (Lynch and Conery 2003; Lynch 2007). The efficacy of this selective force is inversely related to effective population size, while the intensity of the force itself is directly proportional to the mutation rate. Thus, genome size should be inversely correlated with each of these two factors (Lynch 2007; Knibbe et al. 2007).

Both theories receive support from some observations (Yi and Streelman 2005; Kelkar and Ochman 2012; Smith et al. 2013; Sung et al. 2016; Mueller and Jockusch 2018; Canapa et al. 2015; Luiselli et al. 2024) but are also challenged by others (Ai et al. 2012; Sloan et al. 2012; Mohlhenrich and Mueller 2016; Marino et al. 2025). Importantly, they are not mutually exclusive, as a combination of mutational biases and second-order selective effects due to the mutational liability of nonfunctional DNA could act together to determine an equilibrium genome size. This calls for an integrated explanation for what determines the amount and variation of noncoding DNA in genomes. In this direction, previous studies suggest that structural mutations, *i.e.* chromosomal rearrangements or more generally any mutation larger than 50 bp, could be a key element of genome size evolution (Adams et al. 2023; Banse et al. 2024; Luiselli et al. 2024). They could also link both the MEH and the MHH, as structural mutations significantly affect genome size and are also a huge mutational liability in themselves due to their large-scale effect. It is therefore essential to examine their impact on genome size evolution.

Here, we propose a minimal probabilistic model of genome evolution, with the following assumptions: (i) genomes are composed of a coding component made of essential genes and a noncoding component that has strictly no phenotypic effect; (ii) mutations occur at random uniformly over the genome. Our analysis of this model reveals nontrivial patterns: (i) structural mutations do not have the same probability of being neutral and this results in a trend towards increasing genome size; (ii) as larger genomes are more susceptible to double-strand breaks—and thus to structural mutations—, changes in genome size change the probability of future, possibly lethal, structural mutations; (iii) this increased risk of having a larger genome modulates the fixation probability of structural mutations in a way that favors deletions over insertions or duplications. Together, these mechanisms ensure a stable evolutionary equilibrium for noncoding genome size. More precisely, the equilibrium noncoding *fraction* depends on the product of the effective population size and mutation rate of a species ($N_{e}\times \mu$), while the noncoding *size* is determined by this product plus the coding architecture of the genome (coding size and distribution). Notably, the equilibrium is a robust outcome of our model, even in the presence of mutational biases towards insertions or deletions: arbitrary mutational biases merely shift the equilibrium.

Altogether, our model integrates key aspects of the MEH and MHH to provide a general mechanistic explanation for genome size evolution. It highlights structural mutations as a major mutational hazard susceptible to driving genome size evolution under general conditions.

## Model and Results

### Model Overview and Existence of an Equilibrium Noncoding Genome Size

To address the question of noncoding genome size evolution, we study the effect of mutations on a population of *N* individuals with simplified, circular genomes.

**Fig. 1.**
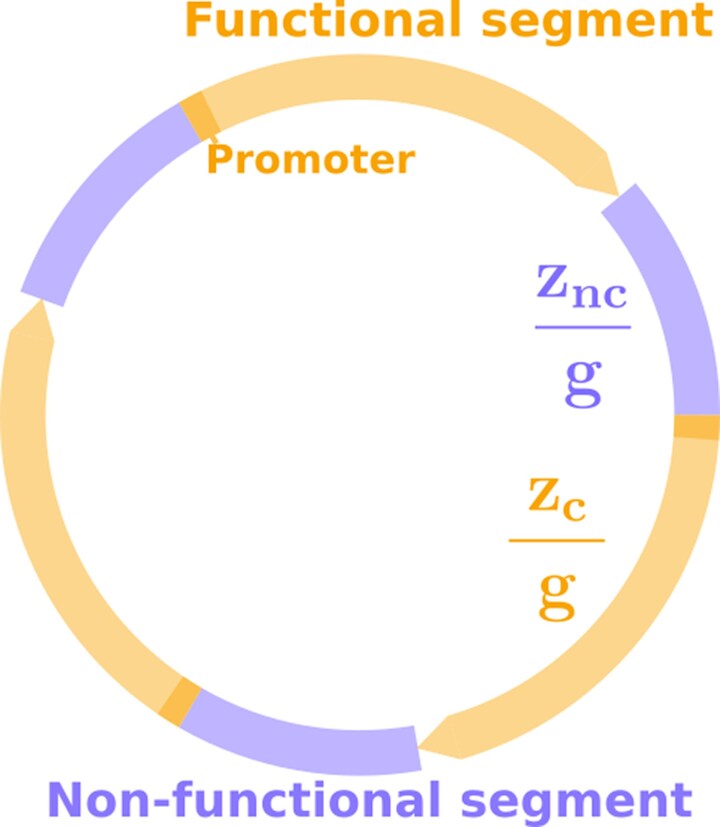
Representation of a genome, with g=3. Noncoding segments are of the same size $z_{\mathrm{nc}}/g$, and coding segments are of the same size $z_{c}/g$. Each coding segment starts with a promoter that can create a new coding segment if duplicated.

As shown in Fig. 1, we consider a circular haploid genome of length *L* base pairs (bp), composed of *g* coding segments (and thus *g* noncoding segments). Let us note the number of noncoding base pairs $z_{\mathrm{nc}}$ and the number of coding base pairs $z_{c}$. We have $z_{c}+z_{\mathrm{nc}}=L$. We assume that:

- Coding segments are of the size $\frac{z_{c}}{g}$ and represent “genes”. Genes are nonoverlapping and all oriented in the same direction. The noncoding segments are equally distributed between the *g* genes and are each of size $\frac{z_{\mathrm{nc}}}{g}\;⩾\;0$. We assume this remains true after any change in noncoding size, as neutral inversions will reshuffle the genome. This ensures that a genome can be fully described with just *g*, $z_{c}$, and $z_{\mathrm{nc}}$.
- Deleting any base of a gene inactivates it and is always lethal. Genes are assumed to have a promoter, here represented by their first base. As a result, a partial duplication not including this first base is not expressed and is thus neutral, *i.e.* it does not affect viability, as long as it is not inserted within a gene. Conversely, a partial or complete duplication including the promoter results in a new expressed gene and is lethal, regardless of its insertion point, *i.e.* any gene duplication is assumed to be lethal.
- Nonlethal mutations are assumed to be perfectly neutral for the viability of the individual. Thus, fitness is binary: it is either 1 or 0.

Different types of mutations occur at different mutation rates. We note *μ* the basal per base mutation rate of the organism, and $\lambda_{i}\mu$ is the per base mutation rate for mutation type *i*. Throughout the manuscript, we analyze the evolution of the noncoding genome size $z_{\mathrm{nc}}$, the coding genome size $z_{c}$ and the number of coding segments *g* being fixed due to the viability constraints.

#### Neutral Genome Growth

We compute the probability of different types of mutations to be neutral and fixed in a population of size *N*. In the following, a mutation is said to be neutral when it does not affect the coding genome and thus does not alter the viability of the individual.

For the sake of clarity, we consider here (Sections 2.1.1 and 2.1.2) a simple version of the model including only two types of structural mutations: duplications (dupl) and deletions (del), occurring at the same per bp rate ($\lambda_{\mathrm{dupl}}=\lambda_{\mathrm{del}}=1$). A duplication copies a random segment of the genome and inserts it elsewhere, while a deletion removes a random segment of the genome. The breakpoints are chosen uniformly at random on the genome, such that both mutations have the same size distribution and the expected change of size of the genome upon one mutation is 0.

We calculate the probability *ν* for each of these two mutations to be neutral (in terms of viability), recalling that duplicating a promoter, inserting a segment within a gene, or deleting any base of a gene is always deleterious. Detailed computations are provided in Supplementary Information (Section S1).


(1) $$\begin{array}{rl} \nu_{\mathrm{del}}(g,z_{c},z_{\mathrm{nc}}) & =\frac{z_{\mathrm{nc}}(z_{\mathrm{nc}}+g)}{2gL^{2}}\\ \nu_{\mathrm{dupl}}(g,z_{c},z_{\mathrm{nc}}) & =\frac{\left(z_{\mathrm{nc}}+z_{c}-g)(z_{\mathrm{nc}}+g\right)}{2gL^{2}}. \end{array}$$

**Fig. 2.**
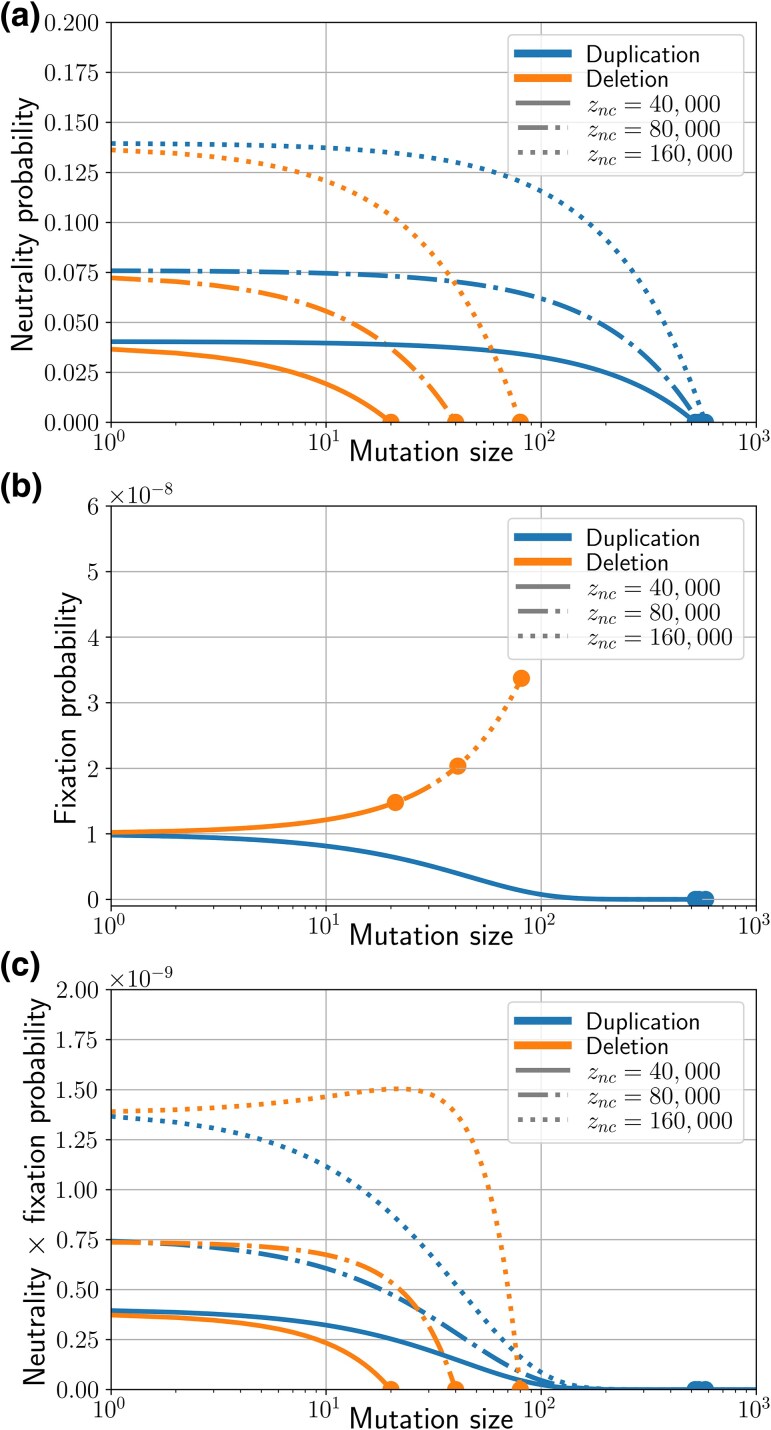
Effect of mutation type and genome size on neutrality and fixation. The dots mark points above which mutations cannot be neutral nor fixed due to constraints from the genome architecture. a) Probability of a duplication (blue) or a deletion (orange) not affecting the coding genome for different mutation sizes and noncoding sizes (Supplementary Information, section S10). The number of genes *g* is fixed at 2,000, and the coding genome size at $z_{c}=1,000,000$ bp. Mutations are more likely to be neutral in bigger and more noncoding genomes, and neutral duplications are larger and more frequent than neutral deletions. b) Probability of fixation of a neutral duplication (blue) or a neutral deletion (orange) for different mutation sizes and noncoding sizes. The number of genes *g* is fixed at 2,000, the coding genome size at $z_{c}=1,000,000$ bp, the population size is $N=10^{8}$, and the mutation rate is $\mu =10^{-10}$ for each mutation type. c) Probability of being neutral *and* fixed for different mutation sizes and different noncoding sizes. The number of genes *g* is fixed at 2,000, the coding genome size at $z_{c}=1,000,000$ bp, the population size is $N=10^{8}$, and the mutation rate is $\mu =10^{-10}$ for each mutation type. For the shortest noncoding size (solid line), duplications are more often neutral and fixed than deletions for any mutation size, indicating that the noncoding genome size would increase. On the contrary, for the biggest noncoding size (dotted line) deletions are more often neutral and fixed than duplications for any mutation size, indicating that the noncoding genome size would decrease: there must be an equilibrium noncoding size between these values.

Notably, we have $\frac{\nu_{\mathrm{del}}(g,z_{c},z_{\mathrm{nc}})}{\nu_{\mathrm{dupl}}(g,z_{c},z_{\mathrm{nc}})}=\frac{z_{\mathrm{nc}}}{z_{\mathrm{nc}}+z_{c}-g}\;⩽\;1$, as $z_{c}$ is obviously much larger than *g*. Thus, duplications are more often neutral than deletions. Similarly, we show that neutral duplications are also on average larger than neutral deletions (see Supplementary Information Section S9). As illustrated by Fig. 2a, we can also consider the probability for a mutation of a given size *k* to be neutral. It highlights that increasing $z_{\mathrm{nc}}$ increases both the probability for mutations of a given size to be neutral and the range of possible neutral mutations (note that, above a certain size, mutations are always lethal due to constraints from the genome architecture: larger mutations would necessarily delete part of a gene or duplicate a promoter). Consequently, genomes should grow indefinitely if we assume that only neutral mutations are fixed with an equal probability. However, a mutation that is neutral in terms of fitness for the individual is not necessarily neutral in terms of fitness for the lineage. The next section will explore the effect of this second-order selective force.

#### Robustness Selection

**Fig. 3.**
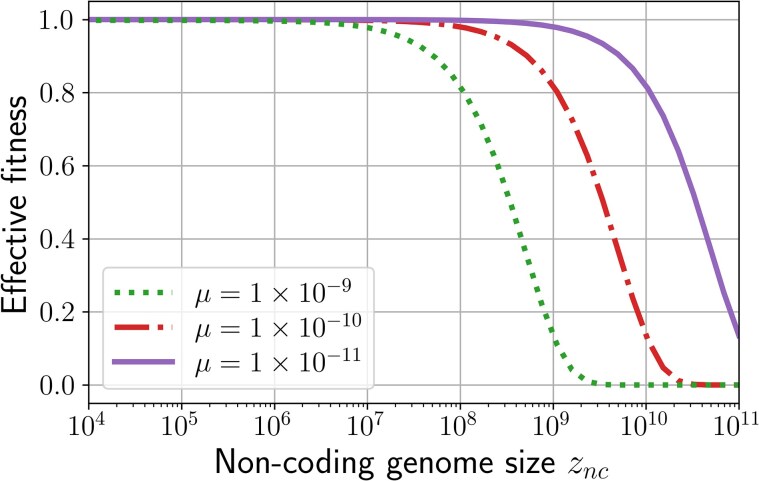
Effective fitness $f_{e}$ for different noncoding sizes $z_{\mathrm{nc}}$ and different mutation rates *μ* (note that *μ* denotes the mutation rate per type of mutation, hence when $\mu =1/(z_{\mathrm{nc}}+z_{c})$ on average one deletion and one duplication occur at each reproduction). Genome architecture is fixed at $z_{c}=1,000,000$ and g=2,000, and $\lambda_{\mathrm{del}}=\lambda_{\mathrm{dupl}}=1$. Notably, the effective fitness decreases with both $z_{\mathrm{nc}}$ and *μ*.

By definition, a neutral duplication or deletion does not change the viability of an individual. However, it changes the noncoding genome size $z_{\mathrm{nc}}$. Now, as shown by Equation (1), the probability for a mutation to be neutral depends on $z_{\mathrm{nc}}$, and so a neutral mutation changes the probability for future mutations to also be neutral. Changing the genome size also changes the probability for a mutation to occur at replication, as bigger genomes will naturally undergo more mutations for the same per base mutation rate. Therefore, mutations that are neutral in terms of their immediate effect on the viability still change the probability for the individual to have future offspring that are equally fit—their robustness. For the rest of the manuscript, we call the *effective fitness $f_{e}$* of an individual the average fitness of its potential offspring. This can also be viewed as the fecundity of an individual once the viability of the offspring has been taken into account. $f_{e}$ can be computed as the probability that, at each position and for each mutation type, either no mutation occurs or the mutation is neutral. As we focus on neutral mutations (with any nonneutral mutation assumed lethal), we consider mutations to be independent. Of note, this is an approximation. In particular, it ignores the mechanistic interference that would in principle occur between rearrangements that overlap in their span over the wild-type genome. However, this approximation is reasonable, considering that we are concerned only with neutral rearrangements, whose rate per genome is typically much less than 1 in all known empirical regimes, so that the probability of multiple neutral events is negligible.


(2) $$\begin{array}{rl} \;f_{e}(\mu ,g,z_{c},z_{\mathrm{nc}}) & =P(\text{all duplications are neutral})\\ & \;\times P(\text{all deletions are neutral})\\ & =\prod_{i=1}^{L}[(1-\mu )+\mu \nu_{\mathrm{dupl},i}(g,z_{c},z_{\mathrm{nc}})]\\ & \;\times \prod_{i=1}^{L}[(1-\mu )+\mu \nu_{\mathrm{del},i}(g,z_{c},z_{\mathrm{nc}})] \end{array}$$

with $\nu_{\mathrm{dupl},i}(g,z_{c},z_{\mathrm{nc}})$ (*resp.*  $\nu_{\mathrm{del},i}(g,z_{c},z_{\mathrm{nc}})$) the probability for a duplication (*resp.* a deletion) starting at position *i* to be neutral, given the genome structure. Both probabilities can be computed using the same approach as for Equation (1) (see Supplementary Information S2) and Fig. 3 shows that the effective fitness $f_{e}$ actually decreases as the noncoding genome size increases—mostly due to the increase in number of mutations as genome size increases. Consequently, selection acts against the genome size increase described in Section 2.1.1.

We characterize this effect more precisely using a population genetics argument. We consider a haploid population of wild-type individuals of size *N* in which a mutant appears and bears a neutral mutation that adds *k* bases to its noncoding genome, with $k\in Z^{*}$. *k* can be either positive (duplication) or negative (deletion). We consider that the population follows a Wright–Fisher model (Fisher 1923; Wright 1931), and we compute the probability for this mutant to go to fixation (Sella and Hirsh 2005):


(3) $$P_{\mathrm{fix}}(k,\mu ,N,g,z_{c},z_{\mathrm{nc}})=\frac{1-{\left(\frac{\;f_{e}(z_{\mathrm{nc}})}{\;f_{e}(z_{\mathrm{nc}}+k)}\right)}^{2}}{1-{\left(\frac{\;f_{e}(z_{\mathrm{nc}})}{\;f_{e}(z_{\mathrm{nc}}+k)}\right)}^{2N}},$$

where we note $f_{e}(\mu ,g,z_{c},z_{\mathrm{nc}})$ simply $f_{e}(z_{\mathrm{nc}})$, as we consider that other parameters are fixed. As illustrated by Fig. 2b, mutations that increase genome size are less likely to be fixed than mutations that decrease genome size. This is the direct consequence of an increase in genome size being tied to an increase in the per genome mutation rate, and hence a decrease in effective fitness (see Fig. 3). Hence, while neutral duplications are more frequent and larger than neutral deletions, they are also more rarely fixed. When considering the combination of these two tendencies (the opposing biases in the immediate probability of being lethal and in the ultimate probability of being fixed), we can see that the shortest genomes are more likely to fix neutral duplications while the longest genomes are more likely to fix neutral deletions, as demonstrated in Fig. 2c. This intuitively results in an equilibrium genome size at which the two effects cancel out.

#### Computing the Equilibrium Noncoding Genome Size

To formalize this equilibrium genome size, we compute the average contribution of duplications ($\delta_{\mathrm{dupl}}$) and deletions ($\delta_{\mathrm{del}}$) to changes in noncoding genome size in the population. $\delta_{\mathrm{dupl}}$ and $\delta_{\mathrm{del}}$ are expressed in bp per generation per mutation event and represent the average length of fixed mutations per time unit. They are computed under the origination–fixation approximation, meaning there is no clonal interference, and we consider the probability for each mutation individually to go to fixation in the absence of any other mutant in the population. Each *δ* thus depends on the mutation’s probability of being neutral, its size, and its fixation probability:


(4) $$\begin{array}{rl} & \delta_{\mathrm{dupl}}(\mu ,N,g,z_{c},z_{\mathrm{nc}})\\ & \;=\frac{g(z_{\mathrm{nc}}+g)}{L^{3}}\sum_{\;j=1}^{\frac{z_{\mathrm{nc}}+z_{c}}{g}-1}\left(\frac{z_{\mathrm{nc}}+z_{c}}{g}-j\right)jP_{\mathrm{fix}}(j)\\ & \delta_{\mathrm{del}}(\mu ,N,g,z_{c},z_{\mathrm{nc}})\\ & \;=\frac{g}{L^{2}}\sum_{\;j=1}^{z_{\mathrm{nc}}/g}\left(\frac{z_{\mathrm{nc}}}{g}-j+1\right)jP_{\mathrm{fix}}(-j), \end{array}$$

where we denote $P_{\mathrm{fix}}(k,\mu ,N,g,z_{c},z_{\mathrm{nc}})$ as $P_{\mathrm{fix}}(k)$, as other parameters are supposed fixed. Detailed derivations are presented in the Supplementary Information, Section S3. From Equation (4), we can derive the bias towards increasing or decreasing genome size as the ratio between the sum of the contributions of all deletions over the sum of the contributions of all duplications for a given genome size, population size, and mutation rate.


(5) $$\begin{array}{rl} B(\mu ,N,g,z_{c},z_{\mathrm{nc}}) & =\frac{\mu \;L\;N\;\delta_{\mathrm{del}}(\mu ,N,g,z_{c},z_{\mathrm{nc}})}{\mu \;L\;N\;\delta_{\mathrm{dupl}}(\mu ,N,g,z_{c},z_{\mathrm{nc}})}\\ & =\frac{\delta_{\mathrm{del}}(\mu ,N,g,z_{c},z_{\mathrm{nc}})}{\delta_{\mathrm{dupl}}(\mu ,N,g,z_{c},z_{\mathrm{nc}})}. \end{array}$$

**Fig. 4.**
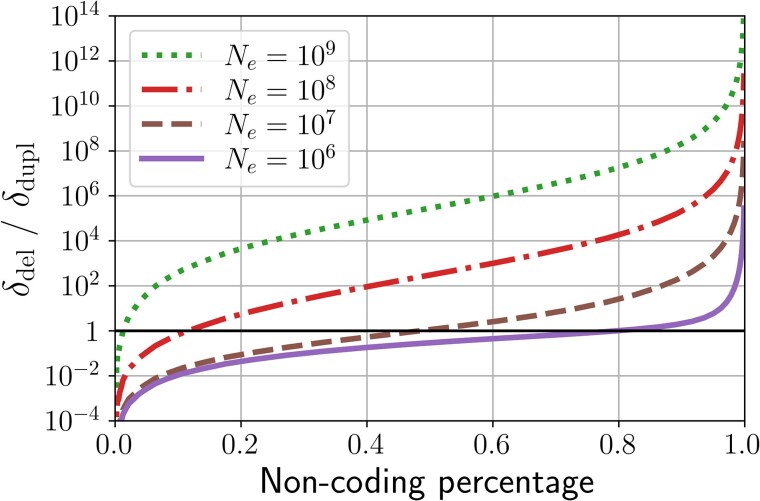
Measured bias for different noncoding proportions. Genome architecture is fixed at $z_{c}=1,000,000$ and g=2,000, the mutation rate is fixed at $\mu =1\times 10^{-10}$ and $\lambda_{\mathrm{del}}=\lambda_{\mathrm{dupl}}=1$. $z_{\mathrm{nc}}$ varies in a logspace from $10^{3}$ to $10^{9}$, and four different values of *N* are depicted, showing a progression in the equilibrium noncoding percentage. The black horizontal line shows the equilibrium at B=1. Supplementary Fig. S1 presents a similar figure with different mutation rates *μ*.

The noncoding genome size is at equilibrium when B=1 (see Fig. 4). When the bias is above 1, deletions contribute more to genome size changes and the noncoding proportion shrinks. On the other hand, when the bias is below 1, duplications contribute more to genome size change and the noncoding proportion increases.

#### Joint Impact of Population Size and Mutation Rate

*B* is a function of the genome architecture ($z_{c}$, $z_{\mathrm{nc}}$, and *g*), the population size *N*, and the mutation rate *μ*. However, we can show that *B* depends on *N* and *μ* only through their product, as previously observed in simulation data (Luiselli et al. 2024).

Indeed, *N* and *μ* only appear in $f_{e}$ (Equation (2)) and $P_{\mathrm{fix}}$ (Equation (3)). Let us start with the expression of the effective fitness $f_{e}$ (Equation (2)):


$$\begin{array}{rl} \;f_{e}(z_{\mathrm{nc}}) & =P(\text{all duplications are neutral})\\ & \;\times P(\text{all deletions are neutral}) \end{array}$$

Using the simplification developed in Supplementary Information (Section S2), and considering that the mutation rate *μ* is negligible compared to 1 (μ≪1), we get:


$$\begin{array}{rl} f_{e}(z_{\mathrm{nc}}) & =(1-\mu {)}^{\left(z_{c}+z_{\mathrm{nc}}\right)}(1-\mu {)}^{\left(z_{c}+z_{\mathrm{nc}}\right)}\\ & \underset{\mu \to 0}{∼}\;\exp\;\left(-2(z_{c}+z_{\mathrm{nc}})\mu \right). \end{array}$$

Then, the ratio of effective fitnesses used in the computation of $P_{\mathrm{fix}}$ (Equation (3)) can be written as:


$$\begin{array}{rl} \frac{\;f_{e}(z_{\mathrm{nc}})}{\;f_{e}(z_{\mathrm{nc}}+k)} & \underset{\mu \to 0}{∼}\frac{\exp\;\left(-2(z_{c}+z_{nc})\mu \right)}{\exp\;\left(-2(z_{c}+z_{nc}+k)\mu \right)}\\ & \underset{\mu \to 0}{∼}\;\exp\;\left(2k\mu \right). \end{array}$$

The probability of fixation of a mutation changing the genome size by *k* (positive or negative) is thus:


$$\begin{array}{rl} P_{\mathrm{fix}}(z_{\mathrm{nc}},k) & =\frac{1-{\left(\frac{\;f_{e}(z_{\mathrm{nc}})}{\;f_{e}(z_{\mathrm{nc}}+k)}\right)}^{2}}{1-{\left(\frac{\;f_{e}(z_{\mathrm{nc}})}{\;f_{e}(z_{\mathrm{nc}}+k)}\right)}^{2N}}\\ & \underset{\mu \to 0}{∼}\frac{1-\exp\;\left(4k\mu \right)}{1-\exp\;\left(4Nk\mu \right)}\\ & \underset{\mu \to 0}{∼}\frac{4k\mu }{1-\exp\;\left(4Nk\mu \right)} \end{array}$$

$P_{\mathrm{fix}}$ appears to be a function of *μ*, and N×μ. Thus, both $\delta_{\mathrm{dupl}}$ and $\delta_{\mathrm{del}}$ can also be written as *μ* times a function of N×μ. Since $B=\frac{\delta_{\mathrm{del}}}{\delta_{\mathrm{dupl}}}$, the *μ*s cancel out and *N* and *μ* always appear in the form of a product in *B*. Given a fixed coding size $z_{c}$ and a number of coding segments *g* (*i.e.* a fixed coding architecture), *N* and *μ* have therefore a similar impact on the equilibrium noncoding size. This can be illustrated by a numerical exploration of the relative effects of *N* and *μ* (Supplementary Information, Section S5).

An alternative way to see this result is by noting that 2kμ is the selection coefficient associated with the effective fitness: $s=\frac{\;f_{e}(z_{\mathrm{nc}}+k)-f_{e}(z_{\mathrm{nc}})}{f_{e}(z_{\mathrm{nc}})}$. Indeed, in the limit μ→0 and $\frac{\;f_{e}(z_{\mathrm{nc}}+k)}{f_{e}(z_{\mathrm{nc}})}\to 1$, and thus s→0, we have $s∼\ln\;\frac{\;f_{e}(z_{\mathrm{nc}}+k)}{f_{e}(z_{\mathrm{nc}})}∼-2k\mu$. The mutation–selection-drift equilibrium, as a general rule, depends only on relative, not absolute, mutation rates (thus here, on the mutational bias). In addition, it depends on the various selective effects implicated in it only through their scaled selection coefficients. Here, Ns∼−2kNμ, and thus, in the end, the mutation–selection-drift equilibrium depends on *N* and *μ* only through their product.

All these results show that an equilibrium noncoding genome size exists and depends on the coding genome architecture (*g* and $z_{c}$) and on the product N×μ.

#### Necessary Condition for the Existence of the Equilibrium

So far, we only considered one type of mutation: structural variations. Other types of mutation change genome size and one could ask whether they would lead to a similar equilibrium. In particular, short indels (<50 bp) can also add or remove bases to the genome and contribute to genome size changes, although less abruptly than structural variants. Most interestingly, if we replicate our model with only short indels (see Supplementary Information Sections S1 and S3), we do not observe an equilibrium genome size. In all situations, $\delta_{\mathrm{inde}l^{+}}>\delta_{\mathrm{inde}l^{-}}$ and so, in the absence of a sufficiently strong mutational bias in favor of deletions, indels induce an infinite growth of the noncoding size, confirming previous observations (Banse et al. 2024). Indeed, indels do not create a selection for shorter genomes on their own (see Supplementary Information, Section S6): although they are more numerous as genome size increases, they are also more often neutral due to their size being limited, and so their effect is more likely to be limited to noncoding parts of the genome. On the opposite, structural variations, being driven by double-strand breaks, conserve their mutational liability when noncoding genome size increases. This makes them a necessary component to observe a pervasive genome size equilibrium.

### Expanded Model of Noncoding Genome Size Evolution

#### Expanded Mutational set and Mutational Biases

Although the existence of an equilibrium specifically requires the presence of structural mutations, other types of mutations, with possibly different mutation rates, could contribute to genome size—directly (by changing the amount of noncoding sequences) or indirectly (due to their intrinsic mutational liability). To account for this, we added four types of mutations to our mathematical model: point mutations (pm), inversions (inv), small insertions (${\mathrm{indel}}^{+}$), and small deletions (${\mathrm{indel}}^{-}$). Each mutation type *i* has its own mutation rate $\lambda_{i}\mu$, and indels have a random size drawn uniformly between 1 and $l_{m}$. The probability of being neutral for all these mutations, and the average contribution to changes in genome size for indels, is presented in the Supplementary Information (Sections S1 and S3).

**Fig. 5.**
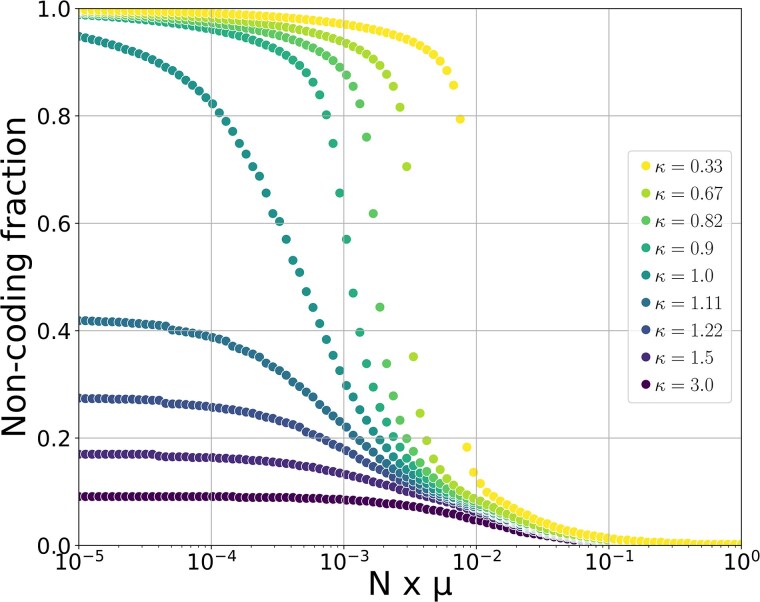
Predicted noncoding fractions for different values of N×μ using the expanded version of the model with six types of mutations. Two sets of equilibrium percentages were run: with $\mu =10^{-9}$ and *N* varying from $10^{4}$ to $10^{9}$, and with $N=10^{8}$ and *μ* varying from $10^{-13}$ to $10^{-8}$. We note the deletion bias $\kappa =\frac{\lambda_{\mathrm{del}}}{\lambda_{\mathrm{dupl}}}=\frac{\lambda_{\mathrm{inde}l^{-}}}{\lambda_{\mathrm{inde}l^{+}}}$. Note that we fix $\lambda_{\mathrm{del}}=\lambda_{\mathrm{inde}l^{-}}$, $\lambda_{\mathrm{dupl}}=\lambda_{\mathrm{inde}l^{+}}$, and these four $\lambda_{i}$ sum to 4. Other parameters are fixed at g=2,000, $z_{c}=1,000,000$, $l_{m}=50$, and $\lambda_{\mathrm{pm}}=\lambda_{\mathrm{inv}}=1$.

Naturally, these mutations and biases displace the equilibrium value of our model, as they change both the robustness of the genomes and the probability of removing or adding new bases. However, Fig. 5 shows that genome size (or equivalently the noncoding fraction) is always a decreasing function of Nμ, whatever the underlying mutational bias. Notably, when there is a deletion bias, the noncoding fraction remains bounded for all values of Nμ. The upper bound is the asymptotic value reached when Nμ→0, and it is smaller for more pronounced deletion biases. Thus, in this regime, not all coding fractions can be achieved by varying Nμ. On the other hand, when there is no bias or an insertion bias, the noncoding fraction diverges in the limit Nμ→0, such that arbitrarily large noncoding fractions can be achieved with sufficiently small Nμ. However, there is always an equilibrium value for Nμ>0. In that case, there is a transition from $z_{\mathrm{nc}}\ll z_{c}$ to $z_{\mathrm{nc}}\gg z_{c}$ as Nμ increases. The higher the mutational bias, the steeper the transition between these regimes.

Notably, the addition of new mutations and the variations in the mutational bias do not suppress the existence of the equilibrium, as they do not fundamentally change the mechanisms at stake. The equilibrium is still determined by the product N×μ and the coding genome architecture ($z_{c}$ and *g*), and the variations are always in the same direction: a higher population size or a higher mutation rate is associated with a lower noncoding fraction.

#### Influence of Varying the Number of Genes

Our results show that, given a coding structure (*g* and $z_{c}$), Nμ and the deletion bias *κ* determine the noncoding genome size at equilibrium. We can also explore the impact of varying the number of genes on our predictions. Knowing that gene size is a very conserved feature within the Tree of Life (Xu et al. 2006), we covaried *g* and $z_{c}$ to keep the gene size constant and showed that the predicted noncoding *fraction* is essentially not sensitive to the number of genes, over a reasonable range of values (500 to 200,000, see Supplementary Information, Section S8). This highlights Nμ and *κ* are the main determinants of the noncoding *fraction*, while the number of genes determines the noncoding *size* that corresponds to this proportion.

### Insights from Biological Data

**Fig. 6.**
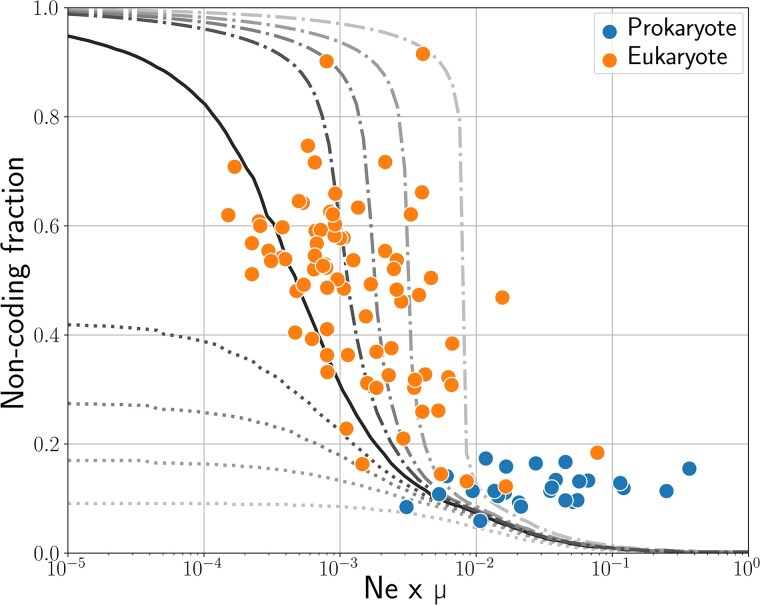
Noncoding fraction plotted against $N_{e}\times \mu$ for 129 species from Lynch et al. (2023). The mutation rate *μ* used here is the per generation per base substitution rate, which we assume to be correlated with the overall mutation rate. The gray lines show the equilibrium noncoding percentages predicted by our model for the same range of N×μ and different mutational biases *κ* (see Fig. 5).

Our model predicts that the *fraction* of the noncoding genome depends only on the compound parameter $N_{e}\;\times \;\mu$, with *μ* the structural mutation rate, as depicted by Fig. 5, as well as on the relative insertion versus deletion rates. The noncoding absolute size has more complicated dependencies, as it also depends on the coding architecture (see Discussion). These predictions could in principle be tested against empirical data. However, spontaneous structural mutation rates are unknown, as most structural mutations are strongly deleterious, hence frequently purged by selection and notoriously difficult to observe and quantify (Ho et al. 2020). Notwithstanding, a tentative comparison with empirical data is shown in Fig. 6, relying on nucleotide diversity to estimate $N_{e}\mu$ and assuming a 1:1 ratio for structural versus point mutation rates (ratios of either 1:10 or 10:1 would shift empirical points to the left or the right, respectively, compared to the theoretical curves).

With a structural mutation rate of this order of magnitude, our model globally predicts the overall trend of the distribution of noncoding fractions observed across species, as a function of nucleotide diversity. Thus, species with a high $N_{e}\times \mu$ present a lower coding fraction than species with a low $N_{e}\times \mu$. More precisely, eukaryotes are mostly located on the left of the figure and have both a higher noncoding fraction and a lower $N_{e}\times \mu$, with a tendency to follow that relationship within them, while prokaryotes are on the right of the figure and present both a lower noncoding fraction and a higher $N_{e}\times \mu$. Notably, the actual noncoding percentage of the prokaryotes shown here is higher than the one predicted by the model. This is expected as our model assumes that the noncoding is purely nonfunctional, while the noncoding genome actually comprises regulatory RNAs and other functional sequences. Thus, this could indicate that most of the “noncoding” base pairs of prokaryotes have a phenotypic effect. Alternatively, this could also be due to incorrect estimations of $N_{e}$ and *μ*, which are notably very hard to measure *in vivo*, or to the simplifications we introduced in our modeling, such as the binary impact of mutations or the uniform distribution of mutation sizes.

Altogether, and even if it is still far from a formal test, this comparison with empirical data gives an idea of the structural mutation rates for which second-order selection on genome rearrangements could represent a key force preventing an infinite genome size growth. It also reproduces the global noncoding genome fraction variation patterns across cellular life. It shows that the potential role of structural mutations in noncoding genome size evolution should not be underestimated and deserves further investigation.

## Discussion

Our model reveals simple yet important evolutionary dynamics on genome size due to two opposite effects. On the one hand, duplications and insertions are more often neutral than deletions, implying a neutral bias towards genome size increase, a mechanism akin to the border selection effect proposed by (He et al. 2019; Loewenthal et al. 2022). As an intuitive example, in the extreme case of a fully coding genome, it is still possible to neutrally insert a base between two genes, while no base can be removed. On the other hand, at constant phenotypical adaptation, bigger genomes are counter-selected as they are more susceptible to structural mutations: lineages in which genomes get bigger are less likely to survive in the long term. This second-order selection for shorter genomes is imposed by the mere existence of structural mutations, which decrease the robustness of genomes when noncoding genome size increases—as previously conjectured (Knibbe et al. 2007). With these two effects, and knowing only the genome’s current coding architecture (size and number of segments), the effective population size of the species, and the rates for the different mutation types, we can determine an equilibrium noncoding genome size towards which the species should be tending. Notably, this does not apply when structural mutations are absent from the model: with only indels, our model predicts that genomes are likely to grow indefinitely.

Naturally, parameters not considered here could impact this equilibrium quantitatively. Most importantly, the presence of transposable elements, noncoding but functional DNA, horizontal transfers, and other mutational processes such as recombination would displace the equilibrium by affecting both the neutral mutational bias and the robustness of genomes. Yet, they would not suppress either of the two above-elaborated effects, and so the existence of the equilibrium would remain—as well as the direction of change in noncoding proportion caused by changes in *N* or *μ*. Similarly, we have assumed a uniform distribution for the sizes of mutations. Relaxing this assumption would also displace the equilibrium, although robustness selection would still operate, as long as the size of the structural mutations increases with genome size—which is intuitive as some species’ structural variants are longer than other species’ genomes (Wellenreuther and Bernatchez 2018). Finally, we have assumed here that any gene duplication is lethal. Although duplicating a gene can be very deleterious (Rice and McLysaght 2017), it is generally expected that this is most often nearly neutral. This could increase the bias towards genome size increase, as inserting bases would be even more neutral, and increase *g*. However, this would not suppress the robustness selection highlighted by the model. Therefore, we expect the two effects we present here to be pervasive. In particular, while the hypotheses of our model are closer to a prokaryote-like genome (a single haploid circular chromosome), there is no reason for the general mechanism to not be true in the case of eukaryotes, and we can use it to compute the predicted noncoding percentage around eukaryote-like values of $N_{e}$ and *μ* (as shown by Fig. 6). To achieve more accurate estimates of equilibrium noncoding genome fractions, one could expand the model to allow for a more precise model of coding segments. In particular, one could distinguish introns and exons within genes—the former being potentially neutrally mutated.

As a first empirical confrontation of our theory, our comparison of the predictions of our model with empirical data (Fig. 6) results in a globally coherent and insightful picture for both eukaryotes and prokaryotes, as predictions could align with biological observations of noncoding fractions. In particular, our model predicts more variability of noncoding fractions in the eukaryotic parameter range, which lies around the steepest part of the curves (see Fig. 6). Conversely, prokaryotes, having a much larger $N_{e}\times \mu$ product, are predicted to be much more stable around lower noncoding fractions. In that case, although a deletion bias in prokaryotes could exist, the $N_{e}\times \mu$ values are in a range where the predicted noncoding percentage is only loosely affected by mutational biases. Mathematically, the high variation of noncoding percentages in eukaryotes could be explained by observing that the function *B* is flatter for lower values of $N_{e}\times \mu$, typical of eukaryotes (see Fig. 4). In these ranges, it is harder to reach the equilibrium noncoding value, as the bias towards losing or gaining bases at each generation is very low, hence allowing more variability of noncoding sizes at constant $N_{e}\times \mu$ and constant mutational bias. Eukaryotes are supposed to be subjected to biases towards insertions (Bingham and Ratcliff 2024 ), making it more complicated to compare data to our model. Finally, the results presented also depend on the rates of different types of mutations, which are largely unknown for structural mutations. Indeed, although they are frequently observed in all domains of life (Raeside et al. 2014; Fang and Edwards 2024), their spontaneous rate is very difficult to estimate due to their strong deleterious effect. As such, interpretations should be taken with caution. In short, our model proposes a possible explanation for the very different noncoding percentages observed in eukaryotes and prokaryotes, without postulating a difference in nature between these two types of organisms, but only relying on the existence of structural mutations and the different values of $N_{e}$ and *μ* for eukaryotes and prokaryotes.

In our parametric exploration, we supposed that *g* and $z_{c}$ covary—a choice justified by the conservation of gene size in the Tree of Life (Xu et al. 2006). In this case, we showed that varying the number of genes has surprisingly little effect on the predicted equilibrium noncoding genome fraction. This startling result can be intuitively explained: increasing *g* increases the bias towards genome size growth (Equation (1)). But increasing *g* also increases $z_{c}$, which decreases the effective fitness (Equation (2)), hence favoring reduced genomes. Strikingly, both effects almost compensate each other in the model (Fig. S4). However, varying independently *g* and $z_{c}$ would probably change the predicted equilibrium noncoding genome fraction by playing differently on these two forces.

Assuming gene size is constant, we showed that the *fraction* of noncoding genome is primarily determined by the parameters $N_{e}\times \mu$ and *κ*. This contrasts with literature data (Petrov 2002; Lynch and Conery 2003; Yi and Streelman 2005; Kelkar and Ochman 2012), that had pointed $N_{e}\times \mu$ and mutational biases as major determinants of (noncoding) *size.* This usual focus on the noncoding size instead of the noncoding genome fraction could explain why some data are not aligned with the MHH (Ai et al. 2012; Sloan et al. 2012; Mohlhenrich and Mueller 2016; Marino et al. 2025). This suggests that further research should focus on noncoding *fractions*, or account for differences in coding architectures (*g*, $z_{c}$) when comparing noncoding genome sizes and $N_{e}$ and *μ* between species.

Finally, although we have assumed fixed mutation rates, in reality, mutation rates are themselves susceptible to evolving. According to the drift barrier model (Sung et al. 2012), mutation rates are under directional selection limited by random drift, such that they reach an evolutionary equilibrium that depends on $N_{e}$. The selective force involved in this higher-order evolutionary process stems from the deleterious effects of new mutations on the offspring of the current generation (Sung et al. 2012; Lynch et al. 2016; Sung et al. 2016). Interestingly, in the case of structural mutations, this force is the same as the second-order selective force behind the mutational hazard (see Equation (2)). Thus, any mutational hazard also represents a selective force acting on modifiers of the corresponding mutation rate. This raises interesting perspectives on the joint evolutionary dynamics of noncoding genome size and structural mutation rates, which would certainly deserve further theoretical exploration.

To conclude, our results show that indirect selection against mutational hazards, *i.e.* robustness selection, and differences in the neutrality of mutations increasing or decreasing genome size are sufficient to explain the existence of an equilibrium in noncoding genome size. Structural mutations are sufficient to fulfill both conditions. The noncoding genome is constantly under indirect selection due to its mutagenic nature and the structural mutations it can initiate following double-strand breaks. As a consequence, a major determinant of the noncoding genome fraction is the product $N_{e}\times \mu$, which affects both the efficacy of selection and the robustness cost of each additional base pair. More research should be conducted into that area to reach quantitative results and to understand more precisely how each determinant of noncoding genome size (*N*, *μ*, mutation bias, types of mutations, number, and length of genes) affects the equilibrium noncoding size of a species, and whether the species are at that equilibrium or tending towards it. Finally, the interaction of indirect selection on the noncoding genome and direct selection on the coding genome should be studied further by relaxing the hypotheses of a fixed coding architecture and a binary fitness.

## Materials and Methods

### Logic behind the Model

We consider a model with a simple genome architecture: organisms own a single circular chromosome. Mutations happen at random on the chromosome, each position for a mutation being drawn from a uniform distribution along the genome. While very simplistic, this approach carries an essential property of structural mutations: their size grows with total genome size (Hirabayashi and Owens 2023). This is easily demonstrated by biological data, as some observed inversions of several Mb (Wellenreuther and Bernatchez 2018) are bigger than the genomes of other organisms. The fact that bigger genomes are more susceptible to mutating and that structural mutations are increasingly dangerous implies a selection for shorter genomes on the lineage level, which we quantify mathematically.

The logic behind the model is the following (detailed computations are provided in the Supplementary Information): the computation of each $\nu_{i}$ is made assuming a uniform draw of all positions needed for the mutation (two for deletions and three for duplications). Each mutation that has any phenotypic effect (deleting part of a gene, duplicating inside a gene, or duplicating a promoter) is lethal. To compute the effective fitness, we consider the probability for each base pair to initiate each type of mutation, and for the initiated mutation to be neutral. By making the approximation that mutations are drawn with replacement, we can compute it as a binomial law where success is defined as either no mutation occurs or a neutral mutation occurs, the number of draws is the genome length multiplied by the number of mutation types (see Supplementary Information, S7.1 for more details). A nonlethal reproduction means there are only successes. The fixation probability is taken from Sella and Hirsh (2005). The average contribution to genome size change of each mutation type is computed as an average of the mutation size weighted by the probability of the mutation to be of this size and by the probability of a neutral mutation of this size to be fixed.

### Numerical Resolution

All the code for the numerical resolution of the mathematical model is available online in GitLab: https://gitlab.inria.fr/jluisell/structural-mutations-set-an-equilibrium-non-coding-fraction. Notably, the code is written in Python using Decimal to increase float precision. This is necessary due to the very large values taken by our parameters (especially genome size and population size) and the several exponents in the computation.

To predict an equilibrium noncoding genome size, we fix the number of genes *g*, the coding genome size $z_{c}$, the effective population size $N_{e}$, the per base per mutation type mutation rate *μ*, and the mutational bias *κ*. We then compute the resulting bias towards adding or losing bases for several possible noncoding sizes. Since the bias function is monotonous with respect to the noncoding genome size, we find the actual equilibrium with a bisect method.

### Biological Data

To compare our model to actual biological data, we gathered mutation rates and effective population sizes from Lynch et al. (2023). For each available species, we downloaded the annotated genome from NCBI and isolated the biggest chromosome. We counted any base pair annotated as part of a protein-coding gene as a “coding” base pair ($z_{c}$), all the other base pairs as “noncoding” ($z_{\mathrm{nc}}$), and we counted the number of continuous noncoding segments (*g*). While more precise annotations could be used (*e.g.* regulatory RNAs could be counted as coding in our model, since they are functional), this approach reduced the annotation bias between well-studied model species and less annotated pioneer species.

## Supplementary Material

msaf315_Supplementary_Data

## Data Availability

The dataset used is derived from sources in the public domain: from NCBI, (https://www.ncbi.nlm.nih.gov/) and from Lynch et al. (2023).
