# Supplementary material for: Structural Mutations Set an Equilibrium Noncoding Genome Fraction
Source: Mol Biol Evol. 2025 Nov 28;42(12):msaf315. doi: 10.1093/molbev/msaf315 (PMC12690204; doi:10.1093/molbev/msaf315)
Supplement: msaf315_Supplementary_Data [file msaf315_supplementary_data.pdf]

Supplementary Information

S1 Probability for a mutation to be neutral

For each type of mutation, we note ν_{mutation} its probability to be perfectly neutral regarding the viability of the individual. We note p_1 the first position uniformly drawn on the genome, and p_2 and p_3 the second and third when needed. As such, each base has a probability $\frac{1}{L}$ to be drawn. There are z_{nc} non-coding bases, distributed along g non-coding segments. Without loss of generality, we assume that the first base of the genome is the start of a coding segment (*i.e.* a promoter). The computations are provided for 6 types of mutations: deletions and duplications, as well as point mutations, small insertions, small deletions, and inversions.

S1.1 Probability for deletions to be neutral

A deletion is neutral if, and only if, the bases deleted are within one of the g non-coding segments. This means that if the first deleted base is at a position i, i must be in the non coding part of the genome, and the last deleted base, at a position j, must be in the same non-coding region. Importantly, since we define the deletion from i to j, due to circularity, if $j < i$, the deleted sequence is the sequence $[i, L]U[1, j]$. This would be deleterious since the first base of the genome is assumed to be a promoter, which would then be deleted.

$$\begin{aligned}\nu_{\mathrm{del}}(g, z_{\mathrm{c}}, z_{\mathrm{nc}}) &= g \sum_{i=1}^{z_{\mathrm{nc}}/g} \left(\frac{1}{L} \sum_{j=i}^{z_{\mathrm{nc}}/g} \frac{1}{L} \right) \\ &= \frac{g}{2L^2} \sum_{i=1}^{z_{\mathrm{nc}}/g} \left(\frac{z_{\mathrm{nc}}}{g} - i + 1 \right) \\ &= \frac{z_{\mathrm{nc}} \left(\frac{z_{\mathrm{nc}}}{g} + 1 \right)}{2L^2}\end{aligned}$$

S1.2 Probability for duplications to be neutral

A duplication is neutral if, and only if, it duplicates a sequence without a promoter basis and copies it at any position in the non-coding regions. The sum over i starts at position 2 to avoid the first base (promoter), and then all duplications are valid as long as they do not encompass the next promoter. This probability is then multiplied by the probability for the insertion point to be in a non-coding region. Note that there are $z_{\mathrm{nc}}/g + 1$ insertion points in a non-coding sequence of size z_{nc}/g as we can insert just before and just after the sequence.

$$\begin{aligned}\nu_{\mathrm{dupl}}(g, z_{\mathrm{c}}, z_{\mathrm{nc}}) &= g \sum_{i=2}^{L/g} \left(\frac{1}{L} \sum_{j=i}^{L/g} \frac{1}{L} \right) \left(g \sum_{k=0}^{z_{\mathrm{nc}}/g} \frac{1}{L} \right) \\ &= \frac{g^2}{L^3} \sum_{i=2}^{L/g} \sum_{j=i}^{L/g} \left(\frac{z_{\mathrm{nc}}}{g} + 1 \right) \\ &= \frac{g(z_{\mathrm{nc}} + g)}{L^3} \sum_{i=2}^{L/g} \left(\frac{L}{g} - i + 1 \right) \\ &= \frac{g(z_{\mathrm{nc}} + g)}{L^3} \sum_{i=1}^{L/g-1} \left(\frac{L}{g} - i \right) \\ &= \frac{g(z_{\mathrm{nc}} + g) \left(\frac{L}{g} - 1 \right) \left(\frac{L}{g} \right)}{2L^3} \\ &= \frac{(z_{\mathrm{nc}} + g) \left(\frac{L}{g} - 1 \right)}{2L^2}\end{aligned}$$

S1.3 Probability for point mutations to be neutral

Point mutations are neutral when they affect a non-coding base, and deleterious when they affect a coding base. The probability to affect a non-coding base is $\frac{z_{\mathrm{nc}}}{L}$:

$$\begin{aligned}\nu_{\mathrm{pm}}(g, z_{\mathrm{c}}, z_{\mathrm{nc}}) &= g \sum_{i=1}^{z_{\mathrm{nc}}/g} \frac{1}{L} \\ &= \frac{z_{\mathrm{nc}}}{L}\end{aligned}$$

S1.4 Probability for small insertions to be neutral

Regardless of their size, small insertions are neutral when outside a coding segment, and deleterious when within a coding segment. Note however that there are $z_{\mathrm{nc}}/g+1$ insertion points in a non-coding sequence of size z_{nc}/g as we can insert just before and just after the sequence.

$$\begin{aligned}\nu_{\mathrm{indel}^+}(g, z_{\mathrm{c}}, z_{\mathrm{nc}}) &= g \sum_{i=0}^{z_{\mathrm{nc}}/g} \frac{1}{L} \\ &= \frac{(z_{\mathrm{nc}}+g)}{L}\end{aligned}$$

S1.5 Probability for small deletions to be neutral

The maximum size l_m of indels events is a parameter of the model. Here, we assume that $z_{\mathrm{nc}}/g \geq l_m$. The rationale here is to calculate the probability of a neutral deletion by separating all non-coding sequences into the $z_{\mathrm{nc}}/g-(l_m-1)$ first bases that can witness deletions of size up to l_m, and the l_m-1 other bases for which only a subset of the possible deletions are neutral. Since the length of the deletion is uniformly chosen between 1 and l_m, if the deletion starts from a basis i close to the end z_{nc}/g of the non coding zone, the probability that it is neutral is $\sum_{k=i}^{z_{\mathrm{nc}}/g} \frac{1}{l_m}$ when $z_{\mathrm{nc}}/g-i<l_m$

$$\begin{aligned}\nu_{\mathrm{indel}^-}(g, z_{\mathrm{c}}, z_{\mathrm{nc}}) &= g\left(\sum_{i=1}^{z_{\mathrm{nc}}/g-(l_m-1)} \frac{1}{L} + \sum_{i=z_{\mathrm{nc}}/g-(l_m-2)}^{z_{\mathrm{nc}}/g} \frac{1}{L} \sum_{k=i}^{z_{\mathrm{nc}}/g} \frac{1}{l_m}\right) \\ &= \frac{g}{L}\left(\frac{z_{\mathrm{nc}}}{g} - (l_m-1) + \frac{1}{l_m} \sum_{z_{\mathrm{nc}}/g-(l_m-2)}^{z_{\mathrm{nc}}/g} \frac{z_{\mathrm{nc}}}{g} - i + 1\right) \\ &= \frac{1}{L}\left(z_{\mathrm{nc}} - g(l_m-1) + \frac{g}{l_m} \sum_{i=1}^{l_m-1} i\right) \\ &= \frac{1}{L}\left(z_{\mathrm{nc}} - g\frac{l_m-1}{2}\right)\end{aligned}$$

S1.6 Probability for inversions to be neutral

An inversion is neutral if the two breakpoints are outside coding regions. Note that the second breakpoint must be different from the first for an inversion to occur. The probability of the inversion to be neutral is thus the product of the two probabilities.

$$\begin{aligned}\nu_{\mathrm{inv}}(g, z_{\mathrm{c}}, z_{\mathrm{nc}}) &= \frac{(z_{\mathrm{nc}}+g)}{L} \times \frac{(z_{\mathrm{nc}}+g)-1}{L-1} \\ &= \frac{(z_{\mathrm{nc}}+g)(z_{\mathrm{nc}}+g-1)}{L(L-1)}\end{aligned}$$

S2 Effective fitness in the simplified model

We consider here a simplified version of the model including only duplications (dupl) and deletions (del) occurring at the same rate μ (see section S7.1 for the model with the full set of mutations). We compute the effective fitness f_e using two assumptions. The main assumption is that mutations are independent:

$$f_e(z_{\text{nc}}) = P(\text{all deletions are neutral}) \times P(\text{all duplications are neutral})$$

This equates to computing all mutations of a given mutant on the same initial genome. This is, of course, an approximation as there is always a non-zero probability that two simultaneous mutations, for instance a deleted segment and a duplicated segment, overlap. However, for the mutation rates considered, the contributions of such probabilities are negligible: in the model, once a lethal mutation has occurred, no subsequent mutation can yield a neutral offspring. Neutral mutations are confined within intergenic regions, and thus the probability of a rearrangement being neutral is at most of the order of $1/g$. Thus, the rate of neutral rearrangement per genome and per generation is at most of the order of $L\mu/g$. In all empirically reasonable parameter regimes, this is small, all the more so as large genomes are generally associated with small mutation rates. Hence, the probability of two rearrangements occurring, being neutral, and overlapping in the same non-coding region is negligible. Thus, the approximation of independence of mutations is reasonable.
We denote m_i the event "a mutation occurs in position i" (and $\neg m_i$ its negation) and n_i "the mutation occurring at position i is neutral. For the sake of clarity, the notation m here is the same for the deletion and for the duplication. We have:

$$\begin{aligned}
P(\text{all possible mutations of type } m \text{ are neutral}) &= \prod_{i=1}^{L} P(\neg m_i \cup (m_i \cap n_i)] \\
&= \prod_{i=1}^{L} [P(\neg m_i) + P(m_i)P(n_i|m_i)] \\
&= \prod_{i=1}^{L} [(1-\mu) + \mu P(n_i|m_i)] \\
&= \prod_{i=1}^{L} [(1-\mu) + \mu \nu_{m,i}]
\end{aligned}$$

Where $\nu_{m,i} = P(n_i|m_i)$ is the notation used in the main text. Note that $\nu_m = \frac{1}{L}\sum_i^L \nu_{m,i}$

S2.1 Probability for all possible deletions to be neutral

A deletion is neutral if, and only if, the bases deleted are within one of the g non-coding segments. This means that if the first deleted base is at a position i, i must be in the non-coding part of the genome, and the last deleted base, at a position j must be in the same non-coding region. Importantly, since we define the deletion from i to j, due to circularity, if $j < i$, the deleted sequence is the sequence $[i, L]U[1, j]$. This would be deleterious since the first base of the genome is assumed to be a promoter, which would then be deleted.

$$\begin{aligned}
P(\text{all possible deletions are neutral}) &= \prod_{i=1}^{L} [1 - \mu + \mu \nu_{del,i}] \\
&= \prod_{k=0}^{g-1} \left[(1-\mu)^{(L-z_{nc})/g} \prod_{i=1}^{z_{nc}/g} (1 - \mu + \mu \nu_{del,i+z_c+kL/g}) \right] \\
&= \prod_{k=0}^{g-1} \left[(1-\mu)^{(L-z_{nc})/g} \prod_{i=1}^{z_{nc}/g} (1 - \mu + \mu \sum_{j=i}^{z_{nc}/g} \frac{1}{L}) \right] \\
&= (1-\mu)^{L-z_{nc}} \left[\prod_{i=1}^{z_{nc}/g} (1 - \mu + \mu \frac{z_{nc}/g - i}{L}) \right]^g
\end{aligned}$$

Note that we have $L > z_{nc} > g$, and in biological data $g \gg 1$. This allows to simplify the last line of the computation to $(1-\mu)^L$, because since in the product we have $1 \leq i \leq \frac{z_{nc}}{g}$, then:

$$
\begin{aligned}
1-\mu \leq 1-\mu+\mu\frac{z_{nc}/g-i}{L} &\leq 1-\mu+\mu\frac{z_{nc}}{gL} \\
\Rightarrow (1-\mu)^{z_{nc}/g} \leq \prod_{i=1}^{z_{nc}/g}\left(1-\mu+\mu\frac{z_{nc}/g-i}{L}\right) &\leq \left(1-\mu+\mu\frac{z_{nc}}{gL}\right)^{z_{nc}/g} \\
\Rightarrow (1-\mu)^{z_{nc}} \leq \left[\prod_{i=1}^{z_{nc}/g}\left(1-\mu+\mu\frac{z_{nc}/g-i}{L}\right)\right]^{g} &\leq \left(1-\mu+\mu\frac{z_{nc}}{gL}\right)^{z_{nc}}
\end{aligned}
$$

and the conclusion comes with the fact that $\left(1-\mu+\mu\frac{z_{nc}}{gL}\right)^{z_{nc}} \sim (1-\mu)^{z_{nc}}$. The final value $(1-\mu)^L$ is what we use in the numerical resolution of the model.

S2.2 Probability for all possible duplications to be neutral

A duplication is neutral if, and only if, it duplicates a sequence without a promoter basis and copy it at any position in the non-coding regions. The sum over i starts at position 2 to avoid the first base (promoter), and then all duplications are valid as long as they do not encompass the next promoter. This probability is then multiplied by the probability for the insertion point to be in a non-coding region. Note that there are $z_{\mathrm{nc}}/g+1$ insertion points in a non-coding sequence of size z_{nc}/g as we can insert just before and just after the sequence.

$$
\begin{aligned}
P(\text{all possible duplications are neutral}) &= \prod_{i=1}^{L}[1-\mu+\mu\nu_{dupl,i}] \\
&= \prod_{k=0}^{g-1}\left[(1-\mu)\prod_{i=2}^{L/g}(1-\mu+\mu\nu_{dupl,i+kL/g})\right] \\
&= \prod_{k=0}^{g-1}\left[(1-\mu)\prod_{i=2}^{L/g}(1-\mu+\mu\frac{z_{nc}+g}{L}\sum_{j=i}^{L/g}\frac{1}{L})\right] \\
&= \prod_{k=0}^{g-1}\left[(1-\mu)\prod_{i=2}^{L/g}(1-\mu+\mu\frac{(z_{nc}+g)(L/g-i)}{L^2})\right] \\
&= \left[(1-\mu)\prod_{i=2}^{L/g}(1-\mu+\mu\frac{(z_{nc}+g)(L/g-i)}{L^2})\right]^{g}
\end{aligned}
$$

Note that we have $L > z_{nc} > g$, and in biological data $g \gg 1$. This allows simplifying the last line of the computation to $(1-\mu)^L$, with exactly the same mechanism that we used for deletions. This is the value we use in the numerical resolution of the model.

S3 Expected contribution to genome size change along evolution

For each mutation changing the genome size, we can compute its expected contribution per generation to the average genome size change for a species in terms of base pairs.

S3.1 Contribution of deletions to genome size change

This corresponds to the average size of a deletion weighted by the probability of neutrality times the probability of fixation, as defined in Equation 3.

$$
\begin{aligned}
\delta_{\text{del}}(\mu, N, g, z_c, z_{nc}) &= g \sum_{i=1}^{z_{nc}/g} \left(\frac{1}{L} \sum_{j=i}^{z_{nc}/g} \frac{1}{L} (j-i+1) \mathbb{P}_{fix}(-(j-i+1)) \right) \\
&= \frac{g}{L^2} \sum_{i=1}^{z_{nc}/g} \sum_{j=i}^{z_{nc}/g} (j-i+1) \mathbb{P}_{fix}(-(j-i+1)) \\
&= \frac{g}{L^2} \sum_{i=1}^{z_{nc}/g} \sum_{k=1}^{z_{nc}/g-i+1} k \mathbb{P}_{fix}(-k) \\
&= \frac{g}{L^2} \sum_{k=1}^{z_{nc}/g} \sum_{i=1}^{z_{nc}/g-k+1} k \mathbb{P}_{fix}(-k) \\
&= \frac{g}{L^2} \sum_{k=1}^{z_{nc}/g} \left(\frac{z_{nc}}{g} - k + 1 \right) k \mathbb{P}_{fix}(-k) \\
&= \frac{1}{L^2} \sum_{k=1}^{z_{nc}/g} (z_{nc} - g(k+1)) k \mathbb{P}_{fix}(-k)
\end{aligned}
$$

S3.2 Contribution of duplications to genome size change

Similarly, this corresponds to the average size of a duplication weighted by the probability of neutrality times the probability of fixation, as defined in Equation 3.

$$
\begin{aligned}
\delta_{\text{dupl}}(\mu, N, g, z_c, z_{nc}) &= g \sum_{i=2}^{L/g} \left(\frac{1}{L} \sum_{j=i}^{L/g} \frac{1}{L} (g \sum_{k=0}^{z_{nc}/g} \frac{1}{L} (j-i+1) \mathbb{P}_{fix}(j-i+1)) \right) \\
&= \frac{g(z_{nc}+g)}{L^3} \sum_{i=2}^{L/g} \sum_{j=i}^{L/g} (j-i+1) \mathbb{P}_{fix}(j-i+1) \\
&= \frac{g(z_{nc}+g)}{L^3} \sum_{i=2}^{L/g} \sum_{j=1}^{L/g-i+1} j \mathbb{P}_{fix}(j) \\
&= \frac{g(z_{nc}+g)}{L^3} \sum_{j=1}^{L/g-1} \sum_{i=2}^{L/g-j+1} j \mathbb{P}_{fix}(j) \\
&= \frac{g(z_{nc}+g)}{L^3} \sum_{j=1}^{L/g-1} \left(\frac{L}{g} - j \right) j \mathbb{P}_{fix}(j)
\end{aligned}
$$

S3.3 Contribution of small insertions (InDel$^+$) to genome size change

This corresponds to the average size of a small insertion weighted by its probability of neutrality times its probability of fixation, as defined in Equation 3.

$$
\begin{aligned}
\delta_{\text{indel}+}(\mu, N, g, z_c, z_{nc}) &= g \sum_{i=0}^{z_{nc}/g} \frac{1}{L} \sum_{k=1}^{l_m} \frac{k \mathbb{P}_{fix}(k)}{l_m} \\
&= \frac{(z_{nc}+g)}{L\, l_m} \sum_{k=1}^{l_m} k \mathbb{P}_{fix}(k)
\end{aligned}
$$

S3.4 Contribution of small deletions (InDel$^-$) to genome size change

This corresponds to the average size of a small deletion weighted by its probability of neutrality times its probability of fixation, as defined in Equation 3.

$$\begin{aligned}
\delta_{\text{indel}^-}(\mu, N, g, z_c, z_{nc}) &= g\left(\sum_{i=1}^{z_{nc}/g-(l_m-1)}\left(\frac{1}{L}\sum_{k=1}^{l_m}\frac{k\mathbb{P}_{fix}(-k)}{l_m}\right)+\sum_{i=z_{nc}/g-(l_m-2)}^{z_{nc}/g}\frac{1}{L}\sum_{k=i}^{z_{nc}/g}\frac{(k-i+1)\mathbb{P}_{fix}(-(k-i+1))}{l_m}\right)\\
&= \frac{g}{L\,l_m}\left(\left(\frac{z_{nc}}{g}-(l_m-1)\right)\sum_{k=1}^{l_m}k\mathbb{P}_{fix}(-k)+\sum_{i=z_{nc}/g-(l_m-2)}^{z_{nc}/g}\sum_{j=1}^{z_{nc}/g-i+1}j\mathbb{P}_{fix}(-j)\right)\\
&= \frac{1}{L\,l_m}\left((z_{nc}-g(l_m-1))\sum_{k=1}^{l_m}k\mathbb{P}_{fix}(-k)+\sum_{s=1}^{l_m-1}\sum_{j=1}^{s}j\mathbb{P}_{fix}(-j)\right)
\end{aligned}$$

S4 Impact of μ on the bias towards genome size growth or shrinkage

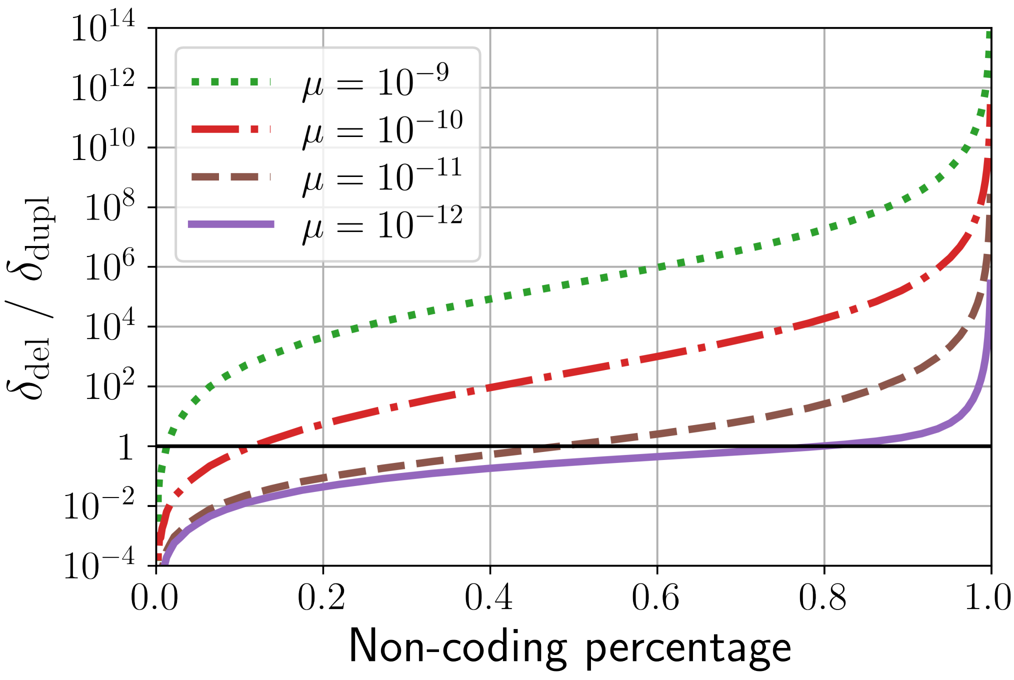

Figure S1. Measured bias for different non-coding proportions and different mutation rates. Genome architecture is fixed at $z_c = 1{,}000{,}000$ and $g = 2{,}000$, the population size is fixed at $N_e = 10^8$ and $\lambda_{del} = \lambda_{dupl} = 1$. z_{nc} varies in a logspace from 10^3 to 10^9, and four different values of μ are depicted, showing a progression in the equilibrium non-coding percentage. The black horizontal line shows the equilibrium at $B = 1$. Note the similarity between this figure and Figure 4 from the main text, where N_e varies instead of μ.

S5 Joined impact of N and μ on non-coding genome fraction at equilibrium

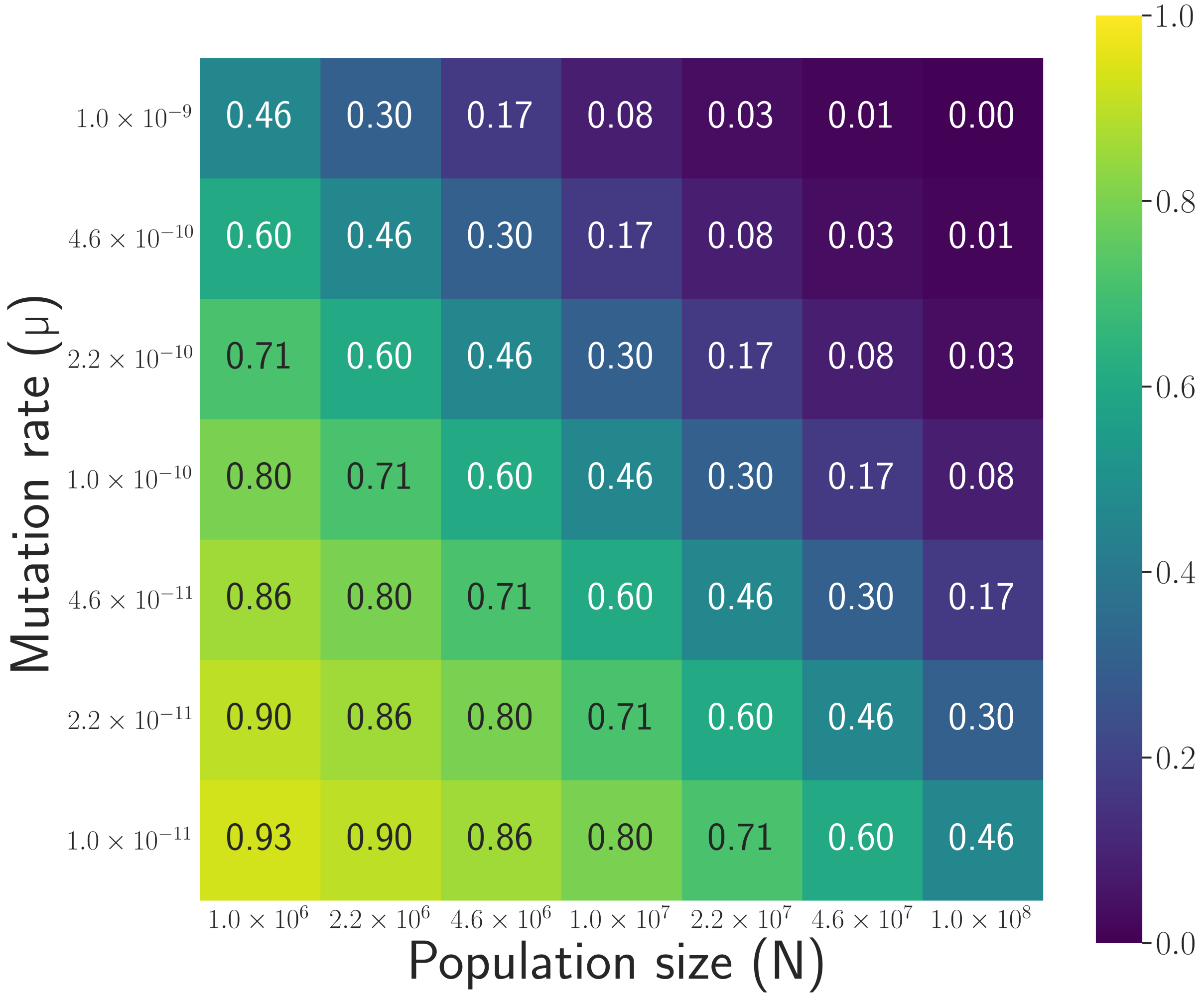

Figure S2. Predicted non-coding fraction at equilibrium for different values of N and μ. The genome architecture is fixed at $z_c = 1{,}000{,}000$ and $g = 2{,}000$, and we have $\lambda_{\text{dupl}} = \lambda_{\text{del}} = 1$.

A change in N or in μ by the same factor results in the same non-coding fraction.

S6 Simplified model with only indels (no structural mutations)

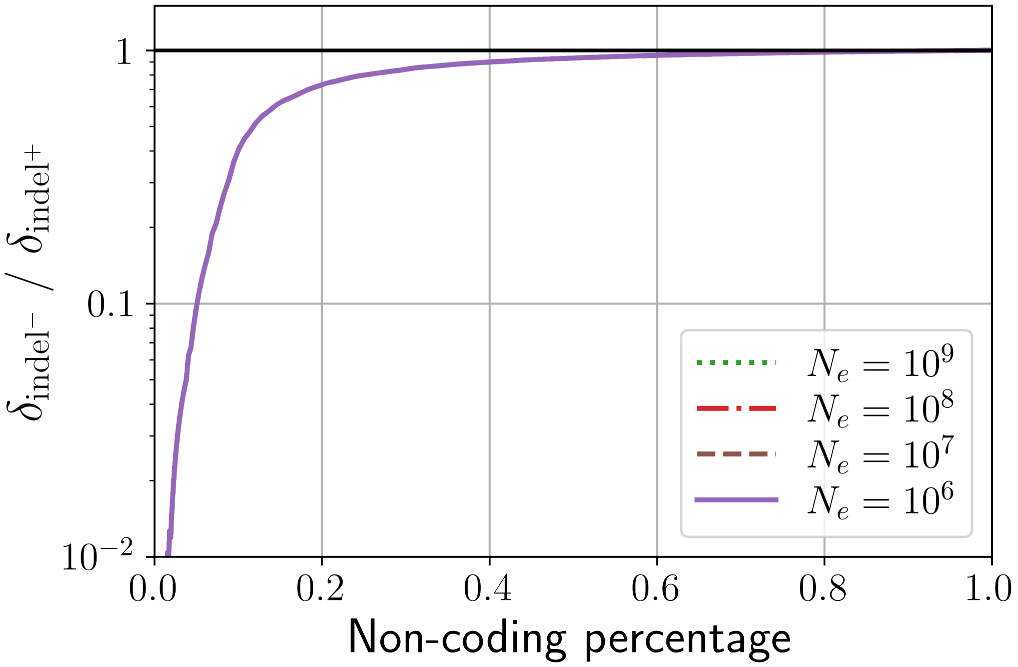

Figure S3. Measured bias for different non-coding proportions. Genome architecture is fixed at $z_c = 1,000,000$ and $g = 2,000$, the mutation rate is fixed at $\mu = 1 \times 10^{-10}$ and $\lambda_{\text{indel}-} = \lambda_{\text{indel}+} = 1$. The maximum size of indels (l_m) is 50. z_{nc} varies in a logspace from 10^3 to 10^9, and four different values of N are depicted. The black horizontal line shows the potential equilibrium at $B = 1$.

Figure S3 shows that the bias converges towards 1 as the non-coding proportion increases, but is always below 1: when only indels are considered, genome size would grow indefinitely in our model.

S7 Equations with the full set of mutations

We note M the set of six mutations: duplications, deletions, inversions, small insertions, small deletions, and point mutations.

S7.1 Effective fitness

We extend the computation of the effective fitness presented in section S2 to account for the other types of mutations.

$$\begin{aligned} f_e(z_{\text{nc}}) &= \prod_{m \in M} P(\text{all mutations of type } m \text{ are neutral}) \\ &= \prod_{m \in M} \prod_{i}^{L} (1 - \mu + \mu \nu_{m,i}) \end{aligned}$$

S7.1.1 Probability for all possible point mutations to be neutral

Point mutations are neutral when they affect a non-coding base, and deleterious when they affect a coding base:

$$\begin{aligned} P(\text{all possible point mutations are neutral}) &= \prod_{i=1}^{L} [1 - \mu + \mu \nu_{pm,i}] \\ &= (1-\mu)^{(L - z_{nc})} \end{aligned}$$

S7.1.2 Probability for all possible small insertions to be neutral

Regardless of their size, small insertions are neutral when outside a coding segment, and deleterious when within a coding segment. Note however that there are $z_{\text{nc}}/g+1$ insertion points in a non-coding sequence of size z_{nc}/g as we can insert just before and just after the sequence.

$$
\begin{aligned}
P(\text{all possible small insertions are neutral}) &= \prod_{i=1}^{L}[1-\mu+\mu\nu_{indel^+,i}] \\
&= (1-\mu)^{(L-z_{nc}-g)}
\end{aligned}
$$

S7.1.3 Probability for all possible small deletions to be neutral

The maximum size l_m of indels events is a parameter of the model. Here, we assume that $z_{\text{nc}}/g \geq l_m$. The rationale here is to calculate the probability of a neutral deletion by separating all non-coding sequences into the $z_{\text{nc}}/g-(l_m-1)$ first bases that can witness deletions of size up to l_m, and the l_m-1 other bases for which only a subset of the possible deletions are neutral. Since the length of the deletion is uniformly chosen between 1 and l_m, if the deletion starts from a basis i close to the end z_{nc}/g of the non coding zone, the probability that it is neutral is $\sum_{k=i}^{z_{\text{nc}}/g} \frac{1}{l_m}$ when $z_{\text{nc}}/g-i<l_m$

$$
\begin{aligned}
P(\text{all possible small deletions are neutral}) &= \prod_{i=1}^{L}[1-\mu+\mu\nu_{indel^-,i}] \\
&= \prod_{k=0}^{g-1}\left[(1-\mu)^{(L-z_{nc})/g}(1)^{z_{nc}/g-(l_m-1)}\prod_{i=1}^{(l_m-1)}(1-\mu+\mu\nu_{indel^+,kL/g-i})\right] \\
&= (1-\mu)^{(L-z_{nc})}\left[\prod_{i=1}^{(l_m-1)}(1-\mu+\mu\sum_{j=1}^{i}\frac{1}{l_m})\right]^g \\
&= (1-\mu)^{(L-z_{nc})}\left[\prod_{i=1}^{(l_m-1)}(1-\mu+\mu\frac{i}{l_m})\right]^g
\end{aligned}
$$

Note that the second part does not depend on L and z_{nc} and can thus be pre-computed.

S7.1.4 Probability for all possible inversions to be neutral

An inversion is neutral if the two breakpoints are outside coding regions. Note that the second breakpoint must be different from the first for an inversion to occur. The probability of the inversion being neutral is thus the product of the two probabilities.

$$
\begin{aligned}
P(\text{all possible inversions are neutral}) &= \prod_{i=1}^{L}[1-\mu+\mu P(n_i|m_i)] \\
&= (1-\mu)^{(L-z_{nc})}(1-\mu+\mu\frac{z_{nc}+g}{L})^{z_{nc}}
\end{aligned}
$$

S7.2 Overall bias

$$
\begin{aligned}
B(\mu,N,g,z_{\text{c}},z_{\text{nc}}) &= \frac{\mu\ L\ N\ \delta_{\text{del}}(\mu,N,g,z_{\text{c}},z_{\text{nc}})+\mu\ L\ N\ \delta_{\text{indel}^-}(\mu,N,g,z_{\text{c}},z_{\text{nc}})}{\mu\ L\ N\ \delta_{\text{dupl}}(\mu,N,g,z_{\text{c}},z_{\text{nc}})+\mu\ L\ N\ \delta_{\text{indel}^+}(\mu,N,g,z_{\text{c}},z_{\text{nc}})} \\
&= \frac{\delta_{\text{del}}(\mu,N,g,z_{\text{c}},z_{\text{nc}})+\delta_{\text{indel}^-}(\mu,N,g,z_{\text{c}},z_{\text{nc}})}{\delta_{\text{dupl}}(\mu,N,g,z_{\text{c}},z_{\text{nc}})+\delta_{\text{indel}^+}(\mu,N,g,z_{\text{c}},z_{\text{nc}})}
\end{aligned}
\quad \textbf{(S1)}
$$

S8 Impact of varying the number of genes

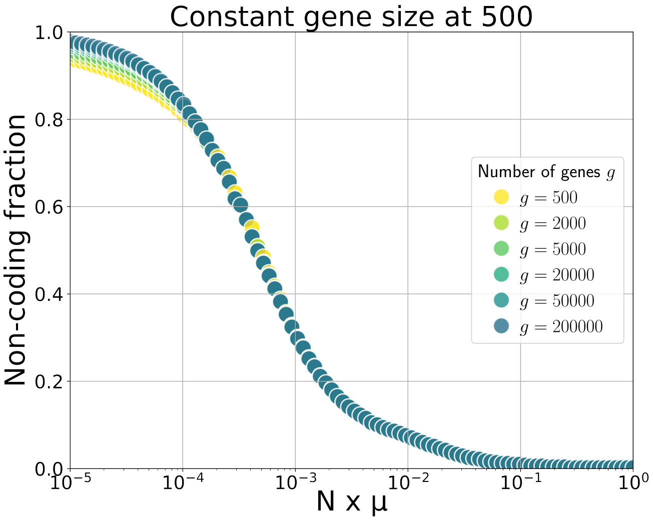

Figure S4. Predicted non-coding fractions for different values of $N \times \mu$ and different number of genes, using the expanded version of the model with six types of mutations. Two sets of equilibrium percentages were run: with $\mu = 10^{-9}$ and N varying from 10^4 to 10^9, and with $N = 10^8$ and μ varying from 10^{-13} to 10^{-8}, with no mutational bias ($\kappa = 1$). The number of genes g here covaries with the coding genome size z_c, such that the gene size is kept constant at $z_c/g = 500$ bp.

We can note that the predicted non-coding *percentage* varies only slightly for different values of g: while the number of genes is a key determinant of non-coding genome *size*, the non-coding *fraction* is still mainly determined by the product $N_e \times \mu$. More exactly, the number of genes has no visible impact on the predicted non-coding fraction when $N \times \mu$ is above 10^{-3} (in which case the model predicts a dense genome with z_{nc} lower than z_c). For lower values of $N \times \mu$, which correspond to genomes predicted to have more non-coding DNA than coding DNA ($z_{nc} > z_c$), an increase in g slightly increases the predicted non-coding percentage, although the effect remains very small.

S9 Average size of spontaneous mutations

We want to compute the spontaneous contribution of the different mutations to genome size changes. As non-neutral mutations are lethal, their size is counted as 0: they cannot change the genome size. Four types of mutations can change the genome size: deletions and duplications, as well as small deletions and small insertions.

S9.1 Genome size change due to a neutral deletion

$$\begin{aligned}
\eta_{del} &= g \sum_{i=1}^{z_{nc}/g} \left(\frac{1}{L} \sum_{j=i}^{z_{nc}/g} \frac{1}{L} (j - i + 1) \right) \\
&= \frac{g}{L^2} \sum_{i=1}^{z_{nc}/g} \sum_{j=i}^{z_{nc}/g} (j - i + 1) \\
&= \frac{g}{L^2} \sum_{i=1}^{z_{nc}/g} \sum_{j=1}^{z_{nc}/g - i + 1} j \\
&= \frac{g}{2L^2} \sum_{i=1}^{z_{nc}/g} (z_{nc}/g - i + 1)(z_{nc}/g - i + 2) \\
&= \frac{z_{nc}(\frac{z_{nc}}{g} + 1)(\frac{z_{nc}}{g} + 2)}{6L^2}
\end{aligned}$$

S9.2 Genome size change due to a neutral duplication

$$
\begin{aligned}
\eta_{\text{dupl}}(g, z_{\text{c}}, z_{\text{nc}}) &= g\sum_{i=2}^{L/g}\left(\frac{1}{L}\sum_{j=i}^{L/g}\frac{1}{L}\left(g\sum_{k=0}^{z_{\text{nc}}/g}\frac{1}{L}(j-i+1)\right)\right)\\
&= \frac{g(z_{\text{nc}}+g)}{L^3}\sum_{i=2}^{L/g}\sum_{j=i}^{L/g}(j-i+1)\\
&= \frac{g(z_{\text{nc}}+g)}{L^3}\sum_{i=2}^{L/g}\sum_{j=1}^{L/g-i+1} j\\
&= \frac{g(z_{\text{nc}}+g)}{2L^3}\sum_{i=2}^{L/g}\left(\frac{L}{g}-i+1\right)\left(\frac{L}{g}-i+2\right)\\
&= \frac{g(z_{\text{nc}}+g)}{2L^3}\sum_{i=1}^{L/g-i}(i)(i+1)\\
&= \frac{g(z_{\text{nc}}+g)\left(\frac{L}{g}-1\right)\left(\frac{L}{g}\right)\left(\frac{L}{g}+1\right)}{6L^3}\\
&= \frac{(z_{\text{nc}}+g)\left(\frac{L}{g}-1\right)\left(\frac{L}{g}+1\right)}{6L^2}
\end{aligned}
$$

S9.3 Genome size change due to a neutral Indel$^-$

A small deletion has size $k \le l_m$ with probability $\frac{1}{l_m}$ so the mean size is :

$$
\begin{aligned}
\eta_{\text{indel}^-}(g, z_{\text{c}}, z_{\text{nc}}) &= g\left(\sum_{i=1}^{z_{\text{nc}}/g-(l_m-1)}\left(\frac{1}{L}\sum_{k=1}^{l_m}\frac{k}{l_m}\right)+\sum_{i=z_{\text{nc}}/g-(l_m-2)}^{z_{\text{nc}}/g}\frac{1}{L}\sum_{k=i}^{z_{\text{nc}}/g}\frac{(k-i+1)}{l_m}\right)\\
&= \frac{g}{L\,l_m}\left(\sum_{i=1}^{z_{\text{nc}}/g-(l_m-1)}\frac{l_m(l_m+1)}{2}+\sum_{i=z_{\text{nc}}/g-(l_m-2)}^{z_{\text{nc}}/g}\sum_{j=1}^{z_{\text{nc}}/g-i+1} j\right)\\
&= \frac{g}{L\,l_m}\left(\frac{l_m(l_m+1)}{2}\left(\frac{z_{\text{nc}}}{g}-(l_m-1)\right)+\sum_{i=z_{\text{nc}}/g-(l_m-2)}^{z_{\text{nc}}/g}\frac{\left(\frac{z_{\text{nc}}}{g}-i+1\right)\left(\frac{z_{\text{nc}}}{g}-i+2\right)}{2}\right)\\
&= \frac{1}{2L}\left((l_m+1)(z_{\text{nc}}-g(l_m-1))+\sum_{j=1}^{l_m-1} j(j+1)\right)\\
&= \frac{1}{2L}\left((l_m+1)(z_{\text{nc}}-g(l_m-1))+\frac{l_m(l_m-1)(2l_m-1)}{6}+\frac{l_m(l_m-1)}{2}\right)\\
&= \frac{1}{2L}\left(z_{\text{nc}}(l_m+1)-g({l_m}^2-1)+\frac{2{l_m}^2-3l_m+1}{6}+\frac{l_m-1}{2}\right)\\
&= \frac{1}{L}\left(z_{\text{nc}}\frac{l_m+1}{2}+g\frac{1-{l_m}^2}{3}\right)
\end{aligned}
$$

S9.4 Genome size change due to a neutral Indel$^+$

$$
\begin{aligned}
\eta_{\text{indel}^+}(g, z_c, z_{nc}) &= g \sum_{i=0}^{z_{nc}/g} \frac{1}{L} \sum_{k=1}^{l_m} \frac{k}{l_m} \\
&= \frac{g}{L\, l_m} \left(\sum_{i=0}^{z_{nc}/g} 1 \right) \left(\sum_{k=1}^{l_m} k \right) \\
&= \frac{(z_{nc} + g)(l_m + 1)}{2L}
\end{aligned}
$$

After calculation, we have $\eta_{\text{indel}^+} > \eta_{\text{indel}^-}$, and since $z_c/g \geq 1$ unless all coding sections are only composed of promoter sequences, we also have $\eta_{\text{dupl}} > \eta_{\text{del}}$. Thus, there exists a neutral bias towards non coding genome size increase. If phenotypical adaptation was constant, genomes would tend to gain more new non-coding bases through duplications than what they lose through deletions. However, we do not observe an infinite growth of genome sizes, and that is due to a variation in the probability of fixation of neutral mutations.

S10 Probability of neutral mutations depending on the size

We compute the probability for a duplication or deletion of size k to be neutral ($\nu_{\text{dupl}}(k)$ and $\nu_{\text{del}}(k)$ respectively).

$$
\begin{aligned}
\nu_{\text{del}}(k) &= \frac{\#(\text{neutral deletions of size } k)}{\#(\text{deletions of size } k)} \\
&= \frac{g \sum_{i=1}^{z_{nc}/g - k} 1}{L} \\
&= \frac{(z_{nc} - kg)}{L}
\end{aligned}
$$

$$
\begin{aligned}
\nu_{\text{dupl}}(k) &= \frac{\#(\text{neutral duplications of size } k)}{\#(\text{duplications of size } k)} \\
&= \frac{\#(\text{positions at a distance at least } k \text{ from a promoter}) \times \#(\text{neutral insertion position})}{\#(\text{first duplicated position}) \times \#(\text{insertion positions})} \\
&= \frac{(L - kg)(z_{nc} + g)}{L^2}
\end{aligned}
$$
